# The effect of multitasking on the communication skill and clinical skills of medical students

**DOI:** 10.1186/s12909-018-1183-5

**Published:** 2018-04-10

**Authors:** Bryony Woods, Aidan Byrne, Owen Bodger

**Affiliations:** 1 Medical School, Cardiff University, Cardiff, UK; 2 Medical School, Swansea University, Swansea, UK

**Keywords:** Mental workload, Clinical skills, Communication skills

## Abstract

**Background:**

Mental workload is an abstract concept that perceives cognition as the brain having a small and finite capacity to process information, with high levels of workload associated with poor performance and error. While an individual may be able to complete two different tasks individually, a combination of tasks may lead to cognitive overload and poor performance. In many high-risk industries, it is common to measure mental workload and then to redesign tasks until cognitive overload is avoided. This study aimed to measure the effect of multitasking on the mental workload and performance of medical students completing single and combined clinical tasks.

**Methods:**

Medical students who had completed basic clinical skills training in a single undergraduate Medical School completed four standardised tasks for a total of four minutes each, consisting of: inactivity, listening, venepuncture and a combination of listening and venepuncture. Task performance was measured using standard binary checklists and with mental workload measured using a secondary task method.

**Results:**

The tasks were successfully completed by 40 subjects and as expected, mental workload increased with task complexity. Combining the two tasks showed no difference in the associated mental workload and performance at venepuncture (*p* = 0.082) However, during the combined task, listening appeared to deteriorate (*p* < 0.001).

**Conclusions:**

If staff are expected to simultaneously complete multiple tasks then they may preferentially shed communication tasks in order to maintain their performance of physical tasks, leading to the appearance of poor communication skills. Although this is a small-scale study in medical students it suggests that the active assessment and management of clinician workload in busy clinical settings may be an effective strategy to improve doctor-patient communication.

## Background

In 2008 Wickens outlined ‘multiple resource theory’ as a way of investigating the effect of increasing workload on the performance of operators in complex environments [1]. This theory defines mental capacity as an abstract concept expressing the maximum rate at which an individual can process information. Mental workload is defined as “the percentage of our mental capacity in use at any one time” [1]. A key prediction of this theory is that if the cognitive demand of any task exceeds the capacity of the operator, then there is an increased risk that the operator will become cognitively overloaded and therefore be prone to error and/or poor performance. The point at which overload occurs is often seen as a balance between three aspects of workload: the demands of the task, the effort exerted by the operator and the expertise of the operator [1]. That is, even where a task is normally completed successfully, performance may deteriorate if the task becomes more difficult, the operator becomes tired, or an expert operator is replaced by a novice.

A review in 2011 found that published studies involving the measurement of the mental workload of clinical staff to be small scale, use widely varying techniques and lack follow up studies to validate their initial findings. However they did identify high mental workload as a common problem, [2] with two areas have a more extensive literature: laparoscopic/robotic surgery [3–11] and the delivery of anaesthesia [12–19]. Within medical education, there are few studies which have used mental workload as an outcome [20–22], although it appears to becoming more common as an outcome in, for example, simulation based training [23].

As mental workload is an abstract concept, it is not possible to measure it directly and a variety of techniques have been developed to provide subjective and objective estimates. Secondary task methods use an additional, simple task completed in addition to the main or primary task. The principle is that the secondary task response will remain constant as long the operator has spare capacity (mental workload < mental capacity). If the workload of the primary task exceeds capacity, there will be no spare capacity to compete the secondary task and secondary task performance will deteriorate [2]. Examples of a secondary task include responding to a vibrating stimulus or performing mental maths, with any delay in response or error used as outcomes [21, 24].

When an operator is required to complete more than one task, the effect on overall workload is may be difficult to predict. Wickens’ multiple resource theory [25] posits that the brain is made up of a variety of different areas which process different types of information. That is, an operator may complete multiple tasks, as long as they do not compete for the same cognitive resource. For example, we may be able to examine a patient (visual) and listen to what they are saying (aural), but we cannot simultaneously listen to a patient (aural) and listen for heart sounds (aural).

Studies in fields such as aviation show that when faced with excessive mental workload, we subconsciously process only some information, so that subjects ignore or ‘shed’ some of the workload in order to maintain their performance in at least one task [26]. Task shedding, can therefore allow an individual to maintain some function in a high workload situation and avoid complete cognitive overload [27, 28].

Many high-risk industries, such as aviation, use mental workload to assess performance, but the techniques are rarely used in medicine, perhaps because the complex nature of the clinical environment makes it hard to define the mental workload of specific clinical tasks [2]. Understanding and measuring mental workload among clinicians is important as excessive mental workload appears to be a common problem within the clinical environment and may be a significant factor in clinical error [29]. A better understanding of the effect of task complexity on mental workload may help us to understand why errors occur and provide new ways of improving patient safety [30].

The aims of this study were to determine whether a series of tasks of increasing complexity would be associated with an increase in measured mental workload and to determine the effect of combining two different tasks on mental workload and performance. A secondary outcome was to further validate the secondary task methodology by measuring mental workload during a period of inactivity.

## Methods

Students studying medicine in 4 different year groups, within a single Undergraduate Medical School, were recruited via social media and poster advertising, with the first 10 volunteers in each year group of 300 students accepted. Data collection took place over a 10 week period. A sample size of ten participants from each year group was chosen empirically as there were no data available to perform a formal power calculation. Only students who had already completed at least one formal teaching session on venepuncture were included. We were not able to identify further opportunities for formal teaching in venepuncture in the later years with students practicing their skills during clinical placements. After ethical approval from the Cardiff School of Medicine Research and Ethics Committee (SMREC ref.: 15/68), forty participants were recruited and provided informed, written consent.

As a secondary task, each participant had an iPhone™ (Apple, Inc., Cupertino, CA, USA), strapped to their non-dominant upper arm by a neoprene phone holder. The iPhone™ used an application called Workload Insight™ (Swansea, UK) programmed to vibrate at random intervals between 10 and 90 s. Participants were asked to tap the screen, as soon as they felt the vibration. Tapping the screen terminated the vibration and the time delay from the onset of the vibration to the response (milliseconds) was recorded by the app.

Each participant completed the same four primary tasks, (Table 1) at the same time as responding to vibration stimuli as a secondary task. All tasks were completed in a single session with only a short break between each task. During Task 1 (control), subjects were asked to sit quietly and to try to achieve a state of zero mental workload. During Task 2, subjects listened to a recorded clinical history for approximately 3 min 15 s, after which participants were asked to answer four factual questions on information contained in the history, scored as 1 for a correct answer, 0.5 for partial and zero for a wrong answer (Appendix 1). During Task 3, subjects were asked to complete a venepuncture on a simulated arm, which included preparation, needle insertion, collection of blood sample and sample labelling. Four minutes were allowed to complete this task and a single observer marked their performance using a 16-point checklist (Appendix 2). All subjects had been trained using the equipment provided and the checklist used was that used by their medical school in summative assessment. During Task 4 subjects completed the listening and venepuncture task at the same time and were assessed on both tasks in the same way. The history provided for the combined task used the same structure and similar questions, but a different history (Appendix 3). The venepuncture task was identical.

**Table 1:** Summary of the four tasks completed by each subject

Task	Activity	Outcomes
1	Control – Inactivity	Mental Workload
2	Listening Task	Mental workload and four post task questions.
3	Venepuncture	Mental workload and 16 point checklist
4	Combined Listening and Venepuncture	Mental workload, 16 point checklist and four post task questions.

Mental workload measurements used a previously validated methodology, with the upper limit of a normal response time as 1350 ms (average + 3SD) [14]. Any response time of less than 1350 ms was regarded as normal with a recorded delay of 0 ms. Any response times greater than 1350 milliseconds were regarded as delayed, with the delay recorded as the response time minus 1350 ms. As described in previous studies, the measure of mental workload used was the average delay, calculated as the total delay in the measurement period, divided by the total number of responses [14].

The normality of distribution of results was determined using a Sharipo-Wilk test. The significance of differences between groups were estimated using Spearman’s rank correlation, with a probability of < 0.05 used as to define significance.

## Results

Forty medical students, a total of 5 males and 5 females from each year, were recruited and all sessions were completed successfully with data available for analysis. Initial analysis of the results showed that neither response time (Sharipo-Wilk, *n* = 160, *p* < 0.0001) nor performance scores (*n* = 160, *p* < 0.0001), were normally distributed, so further analysis used non-parametric methodology.

**Fig. 1:**
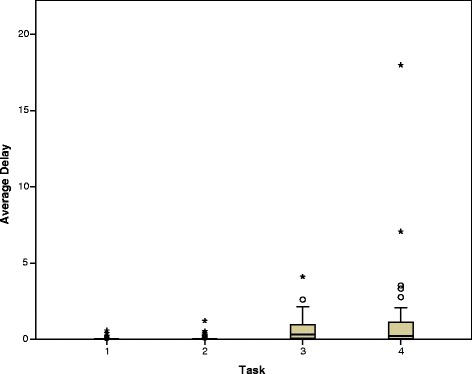
Mental workload expressed as Average Response Delay in seconds during each of the four sequential tasks. (a single value of 17,980 has been excluded from task 4 to aid clarity)

As expected, the average delay in response time increased with the perceived complexity of the task. During Task 1, thirty subjects recorded an average delay of zero consistent with a state of zero mental workload or complete relaxation. The remaining 10 students showed relatively small delays in response times (max 578 ms) consistent with daydreaming or anticipation (Fig. 1). During Task 2, measured mental workload was slightly increased.

During Task 3, subjects showed increased average response times with only 3 subjects having an average delay of zero, with one subject recording an average delay of 4107 ms. Subjects showed similar response times in Task 4 (Fig. 1). Statistical analysis suggested that while there was a significant difference between the first and second pair of tasks, Tasks 1 and 2 were not different, nor were Tasks 3 and 4 (Table 2).

**Table 2:** The statistical significance of pairwise comparisons between the mental workload during different tasks

*p*-values (*N* = 40 in all cases)	Task 1 Control	Task 2 Listening	Task 3 Venepuncture	Task 4 Ven + Listen
Task 1	–	0.398	0.000	0.000
Task 2		–	0.000	0.000
Task 3			–	0.289
Task 4				–

When performing venepuncture on its own (Task 3) subjects scored 13.5 (4.5 - 16) out of 16 (median, range) and 13 (6 - 16) when performing venepuncture at the same time as the listening task (Task 4). The difference was not statistically significant (*n* = 40, *p = 0.082*).

When performing the listening task on its own (Task 2) subjects scored 3 out of 4 (0.5 – 4) and when listening at the same time as venepuncture (Task 4) scored 2 out of 4 (0 – 4). This difference was statistically significant (*n* = 40, *p < 0.0001*).

Subgroup analysis was performed to identify trends in performance between students in different years of study and of different gender, but no significant differences were identified.

## Discussion

This study builds on previous publications using the same methodology and suggests that the secondary task method provides a practical method of measuring the mental workload of those performing clinical tasks [21].

The observed relationship between measured workload and subjective task complexity suggests that it is possible to estimate the mental workload required for specific clinical tasks. However, the nonlinear relationship observed suggests that subjective estimates are inadequate to assign specific levels of workload to individual tasks. As expected, inactivity and listening were confirmed as low workload tasks. The significant rise in average delay during venepuncture however, confirms venepuncture as a high workload task. In particular, the observed level of average delay in some subjects suggests significant cognitive overload consistent with poor performance or error. This was not unexpected as no participant had been formally certified as competent to perform venepuncture and their poor performance was confirmed by the checklist performance scores.

While the performance of venepuncture was expected to be a high workload task, the addition of the listening task was expected to further raise the mental workload of subjects and this was expected to be evident in increased average delay. The observation that the combination of tasks did not increase workload can be explained by two different mechanisms. Firstly, that the subjects improved their level of expertise and were therefore able to perform both tasks with the same level of mental workload (which would seem unlikely given that the tasks were sequential) or that the task itself had changed.

The low measured workload and high listening scores indicate that in the single task the subjects’ had expertise in listening (they were all native speakers of English). The most logical explanation of the significant fall in listening performance in the combined task is that, when faced with cognitive overload, subjects ‘shed’ the listening task [28] and used almost all their available cognitive capacity to complete the venepuncture task. So, while subjects were still overloaded during the combined task, they were at least able to maintain their performance in venepuncture, evidenced by the unchanged checklist scores.

A relevant criticism of this form of research is that the increased response times may simply represent a physical task preventing subjects completing the secondary task. We would reject this hypothesis on two grounds. Firstly, a previous study involving clinical work addressed this question by allowing subjects to identify any period when they were ‘hands full’ and exclusion of these data did not remove the effect of workload on average delay [12]. Secondly, mental workload theory would predict that cognitive overload will impair all aspects of performance including sensory perception, processing as well as motor response. This is supported by previous studies which have shown both delayed response times and impaired recall of events [15, 31].

Research in other fields such as aviation suggest that the process of task shedding in the face of high cognitive workload is an effective mechanism to avoid cognitive collapse [32]. While aviation studies suggest that pilots are more likely to shed tasks perceived as low priority, the processes which direct task shedding are poorly understood [26]. It is therefore not clear whether subjects consciously prioritised venepuncture as the more important task or whether the selection was entirely subconscious. We are not aware of any research which indicates whether physical or verbal tasks are more likely to be shed in response to increased workload and believe that this study provides the first experimental evidence of task shedding during a clinical task.

These data suggest that during clinical tasks, if cognitive workload increases towards capacity, listening tasks may be preferentially shed. Importantly, as the shedding process may be subconscious, the individuals may not be aware that they have effectively stopped listening and missed crucial information. This conclusion is supported by a previous study using the same methodology that has suggested that within simulated consultations, medical student cognitive workload is excessive [29]. Many patients complain about the ability of doctors to listen to their concerns [33] and while communication skills training is an obvious solution, these data suggests that such training may be ineffective if high mental workload is the cause of the failure to listen.

We recognise that this study included only 40 participants, within one UK medical school and that the opportunistic sampling of participants may also have led to self-selection bias. We therefore recognise that these findings may not generalise to other settings. In particular, studies involving clinical staff in a more realistic environment, at different stages in their training would be required to determine whether the problem identified has a significance to clinical practice. We also recognise that randomisation of the tasks would have reduced the effect of any learning during the study period, however, previous studies have demonstrated no learning effect on this particular secondary task method [21].

## Conclusions

This study has confirmed that the secondary task method appears to be a valid measure of cognitive workload during clinical tasks and may be a useful tool to estimate cognitive workload. The most important insight provided by this study is that if individuals are expected to simultaneously engage in multiple tasks, then their performance may be impaired. In particular, communication skills may be particularly susceptible to cognitive overload. While education and training may improve the performance of single tasks, the active assessment and management of clinician workload in busy clinical settings may be an effective strategy to improve doctor-patient communication.

## Appendix 1

### History transcript and questions (task 2)

“It is a Tuesday morning at Newport Gwent Accident and Emergency department. A 30 year old male has come in complaining of abdominal pain that has been present since Sunday morning. It woke him up from his sleep at 5 am. The pain was originally centralised and crampy but since Monday this pain has moved to his right hand side, mainly his right iliac fossa. The nature of the pain is continuous and sharp in nature. It scores 8/10 on the pain scale and the pain is worse on coughing, movement and inspiration. He has only spiked a temperature once – on Sunday and it was 38 °C. He has had no change in bowel habit. He currently feels nauseous and malaised and vomited on Sunday and Monday, once each day. The patient is otherwise fit and well. He had a bad chest infection last month for which he was on antibiotics but he has since made a full recovery. He takes insulin regularly for type 1 diabetes but his control is good and his last HbA1c was satisfactory. He is also highly allergic to penicillin and gets a full anaphylactic reaction if taken. There is a strong family history of bowel cancer for which he undergoes regular screening but his last colonoscopy came back clear. He lives in a house with his wife and two children and works locally as a managing director of a furniture company. He smokes around 10 cigarettes a day due to his stressful job and drinks around 10 units of alcohol steadily over the course of the working week, mainly beer. He mentioned that no-one else in his family has been ill recently and that they are all fit and well. On systems review nothing was flagged up as suspicious although the patient has recently started complaining of diarrhoea. On examination the patient has tenderness and guarding in the right iliac fossa. Rebound tenderness and percussion tenderness were also present. Bowel sounds were audible. The patient had some blood tests taken and the results came back. His white blood cell count is raised, his CRP is raised but his haemoglobin, platelet count and mean corpuscular volume came back normal. When asked if he has had any trouble urinating he explained he has had some mild pain whilst urinating but did not experience any urgency or feelings of incomplete emptying of his bladder. His liver function tests were normal, as were all components within his urea and electrolytes test.”

Questions:

1. What was the age of the patient?
2. What degree Celsius did his temperature spike to?
3. How many cigarettes does he smoke?
4. What 2 blood tests came back raised?

## Appendix 2

**Table 3:** Venepuncture checklist

Number	Checklist item
1	Washes hands and introduces
2	Collects all equipment
3	Applies tourniquet and finds vein
4	Cleans skin overlying vein
5	Allows to dry
6	Correctly identifies bottles
7	Set up needle and vacutainer
8	Insert needle with bevel upwards
9	Push blood sample bottle into vacutainer
10	Change bottle whilst keeping needle in place
11	Remove tourniquet
12	Remove bottle
13	Remove needle
14	Apply pressure with cotton wool
15	Dispose of sharps correctly
16	Label bottles correctly

## Appendix 3

### History transcript and questions (task 4)

“It is a Thursday afternoon in Cathays GP surgery. A 14 year old has come in to see you complaining of fatigue that has been lasting approximately 2 months. She feels weak all the time and is struggling to do a full day at school without feeling completely exhausted. She feels embarrassed as she has to ask to leave class every lesson, if not twice a lesson, to go to the toilet. She has also been drinking approximately 1 L of water every 2 h as she is feeling constantly thirsty. She gets up 2-3 times every night to go to the toilet and downs a glass of water every time she wakes up, as she is so thirsty. Her mum mentions that her daughter has lost approximately 2 kg of weight this past week despite maintaining a good appetite and has had to stop playing her favourite sport lacrosse, as well as all her other sports, over the past few weeks because she has been feeling so tired and under the weather. She also mentions that towards the end of the day she has to sit closer to the front of class because she struggles to see the board as the day goes on. The patient is usually fit and well. She enjoys swimming, netball and lacrosse and is a very active girl, which is why her mother finds this extreme tiredness so out of character. The patient is on the contraceptive pill for bad acne but is not on any other medication. She is not allergic to any medications but is allergic to latex. When she was younger, she had a bad fracture of her tibia and fibula and had to have an operation and an overnight hospital stay but has no other past medical history. There is family history of sudden cardiac death, with her sister dying suddenly at 17 last year. It is thought that this was due to hypertrophic cardiomyopathy. The patient looks tired in herself on examination and is clinically dehydrated. There were no other findings on examination. The GP orders some blood tests which come back with the following results. Her fasting blood glucose is 11.2 mmol/L, but her serum ferritin was 55 and her haemoglobin was 10 mmol/L respectively. Urinalysis was positive for glucose but the patient denies any urgency or pain during urination.”

Questions:

- How much weight has she lost?
- What is she allergic to?
- What is the reason her sister died last year?
- What was her fasting blood glucose level?
